# Comparison of the responsiveness of the WOMAC and the 12-item WHODAS 2.0 in patients with Kashin–Beck disease

**DOI:** 10.1186/s12891-020-03210-8

**Published:** 2020-03-25

**Authors:** Lei Yang, Dimiao Wang, Xiuxia Li, Hangjing Yuan, Hua Fang, Xiong Guo

**Affiliations:** 1 School of Nursing, Health Science Center, Xi’an Jiaotong University, Xi’an, Shaanxi People’s Republic of China; 2 School of Public Health, Health Science Center, Key Laboratory of Trace Elements and Endemic Diseases of National Health and Family Planning Commission, Collaborative Innovation Center of Endemic Diseases and Health Promotion in Silk Road Region, Xi’an Jiaotong University, No.76 Yanta West Road, Xi’an, Shaanxi 710061 People’s Republic of China

**Keywords:** Responsiveness, WOMAC, WHODAS 2.0, Kashin-Beck disease, Measurement properties

## Abstract

**Background:**

Several questionnaires have been used to assess the health status of patients with Kashin-Beck disease (KBD) in clinical trials, but the evidence regarding the responsiveness of these instruments in KBD patients is limited. Therefore, the aim of this study was to evaluate and compare the responsiveness of the Chinese version of the Western Ontario and McMaster Universities Osteoarthritis index (WOMAC) and 12-item World Health Organization Disability Assessment Schedule 2.0 (WHODAS 2.0) in KBD patients undergoing intra-articular injection of hyaluronic acid (HA).

**Methods:**

A sample of 232 KBD patients treated with intra-articular injection of HA completed the WOMAC, 12-item WHODAS 2.0 and joint dysfunction index (JDI) both pre- and post-treatment. Responsiveness was assessed using correlation and receiver operating characteristic (ROC) curve analyses following the COnsensus-based Standards for the selection of health Measurement INstruments (COSMIN) checklist.

**Results:**

Overall, there were significant improvements in the mean scores on the WOMAC and on the 12-item WHODAS 2.0, except for in the cognition domain. Correlation analysis showed that changes in the WOMAC and 12-item WHODAS 2.0 scores had moderate or weak positive associations with the changes in the JDI. However, acceptable areas under the ROC curve (value > 0.7) were found for all domains and for the total score on the WOMAC, but only for the mobility domain and the total score on the 12-item WHODAS 2.0.

**Conclusions:**

These results demonstrated that the WOMAC was more responsive than the 12-item WHODAS 2.0 in KBD patients treated with intra-articular injection of HA. Our findings support the continued use of the WOMAC as an outcome measure in assessing disability in KBD patients.

## Background

Kashin-Beck disease (KBD) is a chronic, progressive and degenerative osteoarticular disorder with an unknown etiology [1]. It is primarily found in individuals in an endemic agricultural region in southeastern Siberia, North Korea and a belt-shaped region from northeastern to southwestern China [2, 3]. The disease has seriously affected the populations in China. In 2017, it was endemic in 379 counties of 13 provinces or autonomous regions, with 0.54 million individuals suffering from KBD, and an estimated 37.2 million individuals at high risk of developing KBD [4].

**Fig. 1:**
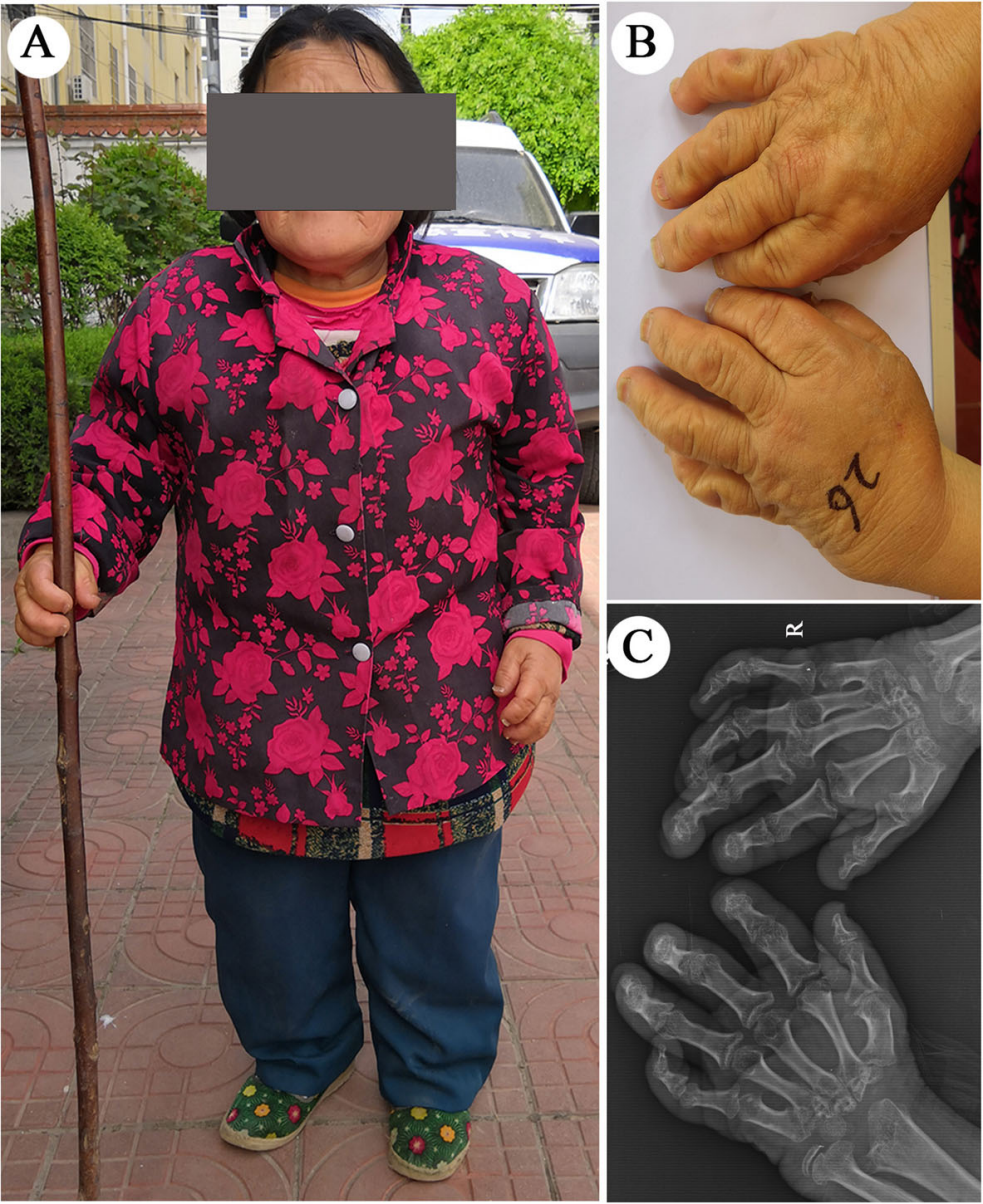
Characteristics of Kashin-Beck disease (KBD). **a** A representative patient severely affected with KBD (female, 57 years old, height 102 cm), which manifested as deformed and shortened fingers, a short stature and limited motion of the joints in the extremities. **b** & **c** Images and radiographic images of the bilateral hands of this patient, showing deformed fingers and metaphyseal lesions in the phalanges

The onset of KBD usually occurs during childhood between 5 and 13 years of age, with the typical initial pathological changes of chondrocyte necrosis in the deep zone of the epiphysis plate cartilage and articular cartilage [5, 6]. Clinically, the destruction of epiphyseal and articular cartilage can result in metaphyseal enlargement, skeletal development impairment and multiple joint deformities. The fingers, toes, wrists, elbows, ankles and knees are the joints that are most often involved, and the affected individuals commonly experience symptoms such as joint enlargement, shortened limbs and fingers (toes), joint pain, morning stiffness, limited motion, and in severe cases, short stature and dwarfism (Fig. 1) [3, 7, 8]. The treatment of KBD is still a major challenge because of the lack of existing clinical guidelines. As adult KBD patients suffer degenerative symptoms that are similar to those experienced by osteoarthritis (OA) patients, the treatment of KBD has been based on the experience of OA treatment. The intra-articular injection of hyaluronic acid (HA) is widely accepted as a treatment for OA. Currently, it has also been shown to be a safe and effective treatment for KBD patients with knee joint involvement [9].

Because daily life and functionality are seriously affected in adult KBD patients with disease progression, a number of tools have been developed and validated to assess the different dimensions of the health status of KBD patients, such as the Western Ontario and McMaster Universities Osteoarthritis index (WOMAC) [10], the joint dysfunction index (JDI) [11] and the 12-item World Health Organization Disability Assessment Schedule 2.0 (WHODAS 2.0) [12]. The WOMAC is a well-known self-administered instrument that was developed for the assessment of hip and knee osteoarthritis [13]. It has been widely used in clinical trials to evaluate the efficacy of intra-articular injection of HA, oral medication, and traditional medical and physical therapies for patients with KBD [14–17]. The WHODAS 2.0 is a generic assessment instrument developed by the World Health Organization (WHO) to measure functioning and disability in major life domains [18]. A recent [12] study showed that the 12-item Chinese version of the WHODAS 2.0 is also a reliable and valid questionnaire for the assessment of disability in KBD patients.

Because they determine important outcome measures, self-reported questionnaires used in clinical studies must be reliable, valid, and responsive [19, 20]. The responsiveness is the ability of an instrument to detect change over time in the construct being measured [21]. It is also considered a vital criterion of an instrument used to evaluate therapeutic effectiveness [22]. However, to the best of our knowledge, few studies have evaluated the responsiveness of outcome measures in KBD patients. Given the increasing use of the WOMAC and 12-item WHODAS 2.0 in clinical research on KBD, there is a need for more detailed evaluations of the responsiveness of these instruments to determine whether it is appropriate to adopt them as outcome measures for clinical KBD interventions. Therefore, we evaluated and compared the responsiveness of the WOMAC and the 12-item WHODAS 2.0 in patients with KBD undergoing intra-articular injection of HA.

## Methods

### Study design and participants

This study was designed as a prospective study and conducted from April 2017 to May 2019 in Shaanxi Province, China. This province has the largest number of patients with KBD in China [4]. Linyou and Yongshou counties were randomly selected from the 62 KBD epidemic counties in Shaanxi Province. We recruited individuals who volunteered to participate in the study with assistance from the Centers for Disease Control and Prevention (CDC) in the two counties. First, all volunteered individuals were asked to undergo X-ray examinations of both the hand and knee joints for the diagnosis of KBD and to assist in the determination of a treatment plan. Then, the diagnosis of KBD was made by a clinician and confirmed by a specialist at the Institute of Endemic Disease, Health Science Center, Xian Jiaotong University according to the clinical and radiological diagnostic criteria for KBD (WS/T 207–2010) (National Health Commission of the People’s Republic of China, http://www.nhc.gov.cn/wjw/s9500/201006/47920.shtml). The four most significant criteria are as follows: (1) radiological changes in the distal end of the bones of the middle and proximal phalanges of the index and ring fingers; (2) focal or irregular premature closure of the epiphysis; (3) limited motion and enlargement of the peripheral joints, deformities and dwarfism; and (4) non-inflammatory lesions in multiple joints.

We included individuals who met the following criteria: (1) diagnosis of KBD with knee joint involvement; (2) aged 18 years or older; (3) residing in a KBD endemic area for longer than 6 months; and (4) capable of reading or understanding Chinese. The exclusion criteria were as follows: (1) had difficulty understanding the questionnaires; (2) had other forms of osteoarthropathy, such as OA, rheumatoid arthritis or recent joint trauma; (3) had received intra-articular injections or surgical treatment of the involved knee joint within the 6 months prior to enrolment; (4) had an allergy to avian products or the drugs to be injected; or (5) suffered from mental illness and other diseases leading to chronic pain or disability that would interfere with the outcome assessments during the study.

All included patients received free treatment with the intra-articular injection of HA (2 ml: 20 mg, Shandong Boshilun Furuida Pharmaceutical Co. Ltd.) in the target knee joint by a well-trained surgeon according to the standard clinical guidelines. The target knee was selected according to the complaints from the patient and the evaluation of the knee joint X-ray performed by the clinician. The dose of HA was 2.0 ml at weekly intervals for a total of 3 weeks. Any patients who experienced serious adverse reactions, including shock symptoms, severe allergic reactions and intra-articular infections, during the treatment were excluded from the study. Baseline demographic and clinical characteristics (such as deformed or enlarged finger joints) were recorded prior to the treatment. Sets of questionnaires were administered before and after the treatment. Considering the low educational background of some participants in this study, the data collection was conducted via one-to-one interviews with five well-trained investigators using a unified method of questioning. This study was approved by the ethics committee of the Health Science Center, Xi’an Jiaotong University, and all included participants provided informed consent before any activity was conducted.

### Assessment questionnaires

The WOMAC is a self-administered, disease-specific instrument that was developed to evaluate disability in patients with knee or hip OA [13]. It is a multidimensional questionnaire consisting of 24 items across three dimensions: pain (5 items), stiffness (2 items) and physical function (17 items). Each question (item) is scored on a 5-point Likert scale ranging from 0 to 4 (none, mild, moderate, severe and extreme). The final score for each dimension was determined by summing the corresponding items, and was normalized to a scale ranging from 0 to 100. A higher score indicates a worse health status, and a lower score indicates a better status. We used the adapted Chinese version of the WOMAC, and the Cronbach’s alpha and intraclass correlation coefficients (ICCs) for the three domains in OA patients ranged from 0.67 to 0.82 and 0.82 to 0.88, respectively [23].

The WHODAS 2.0 is a general measure of functioning impairment and disability in major life domains [18, 24]. It is based on the International Classification of Functioning, Disability and Health (ICF). The complete version of the WHODAS 2.0 contains 36 items that assess the level of functioning in six life domains: (1) cognition; (2) mobility; (3) self-care; (4) getting along; (5) life activities; and (6) participation in society. A 12-item version including two items from each domain has been developed and accounts for 81% of the overall variance in the full 36-item version. It employs a 5-point rating scale for all items, ranging from 0 to 4 (none, mild, moderate, severe and extreme). A complex scoring method was used, in which the scores within each domain and all six domains were summed and converted to an overall score ranging from 0 to 100 (0 = no disability; 100 = full disability). We used the Chinese version of the 12-item WHODAS 2.0 [25], and the Cronbach’s alpha and ICCs for the six domains in KBD patients ranged from 0.70 to 0.91 and 0.69 to 0.85, respectively [12].

The JDI is a recommended tool for assessing therapeutic efficacy in KBD patients (WS/T79–2011) that was developed by the National Health Commission of the People’s Republic of China in 2011 (http://www.nhc.gov.cn/wjw/s9500/201112/53525.shtml). The JDI includes the following five items: arthralgia during nocturnal rest, arthralgia during walking, morning stiffness, maximum distance walked and activities of the lower limb. Each item is scored on a scale ranging from 0 to 2. The total JDI score was determined by summing the scores from all five items. A higher score represents worse joint dysfunction. The improvement rate = (total JDI score before treatment – total JDI score after treatment)/ total JDI score before treatment × 100%. An improvement rate ≥ 70% was considered significantly effective, 30–70% was considered effective, and < 30% was considered ineffective. The Cronbach’s alpha and split-half reliability coefficient of the JDI in KBD patients were 0.69 and 0.68, respectively [11].

### Statistical analyses

All data were entered into Microsoft Office Excel 2016 twice by two independent research assistants and checked for inconsistencies. Then, data analysis was performed using SPSS Statistics software, version 19.0 (IBM, Chicago, IL, USA) by the main investigator with assistance from an independent statistician. The missing values were carefully checked and filled by analysis with SPSS. The descriptive statistics are presented as the mean and standard deviation (SD) for the continuous variables and numbers with percentages for the categorical variables. Paired t-tests were used for the comparison of the scores at baseline and after therapy. Moreover, we assessed the floor and ceiling effects for the WOMAC and the 12-item WHODAS 2.0 because the responsiveness is limited if floor or ceiling effects are present [26]. Floor and ceiling effects for all items were represented using the proportion of the total respondents with the lowest and highest possible scores, respectively. If more than 15% of the respondents have the lowest or highest possible scores, a floor or ceiling effect, respectively, is considered to exist [27]. A *p*-value less than 0.05 was regarded as statistically significant for all tests.

Responsiveness was evaluated according to the COnsensus-based Standards for selection of health Measurement INstruments (COSMIN) recommendations. The JDI was used as the gold standard measure in this study. According to the JDI, patients with an improvement rate ≥ 30% were considered improved, and those with an improvement rate < 30% were considered unimproved (stable or worse) after the treatment. Responsiveness was first evaluated by calculating the correlation coefficients of the change scores pre- and posttreatment across the WOMAC, the 12-item WHODAS 2.0 and the JDI with Spearman’s correlation analysis. Correlation coefficients of 0.25–0.49, 0.5–0.74, and ≥ 0.75 were considered to represent weak, moderate, and strong associations, respectively [28]. Then, we also tested the correlation of the changes between the WOMAC and the 12-item WHODAS 2.0 based on the hypothesis that a moderate positive correlation exists between the changes in the two instruments, which were used to measure the same construct. Moreover, we used receiver operating characteristic (ROC) curves to assess the ability of the two questionnaires to correctly classify patients as improved (improvement rate ≥ 30%) or unimproved (improvement rate < 30%) according to the external anchor. Then, the area under the curve (AUC) was estimated; an AUC value of at least 0.7 was considered to indicate adequate responsiveness [26].

## Results

### Participant characteristics

A total of 249 patients with KBD met the inclusion criteria, provided baseline information and completed the questionnaires before treatment. All of them received the first treatment. Of these patients, 232 (93.2%) received all three treatments and completed the questionnaires after the treatment. The baseline demographics and clinical characteristics of the participants included in the analysis (*n* = 232) are shown in Table 1. The mean age of the patients was 60.75 years, with 54.3% being female. After all three treatments, a total of 15 patients reported adverse reactions (11 reported injection site pain, and 4 reported injection site swelling). All the reported adverse reactions were mild and transient, and these patients experienced symptoms that disappeared or were relieved within one or two days after rest. Moreover, none of the patients reported any serious adverse reactions during the observation period.

**Table 1:** Basic characteristics of the participants (*n* = 232)

Characteristics	Values, n (%) unless stated
Demographics characteristics	
Age (years, mean ± SD)	60.75 ± 6.74
Gender	
Male	106 (45.7)
Female	126 (54.3)
Marital status	
Married	199 (85.8)
Single	3 (1.3)
Widowed	30 (12.9)
Occupation	
Farmer	212 (91.4)
Others	20 (8.6)
Education	
Illiterate	82 (35.3)
Primary school	61 (26.3)
Middle school	60 (25.9)
Higher school	26 (11.2)
University	3 (1.3)
Clinical characteristics	
Deformed finger joints	
Yes	205 (88.4)
No	27 (11.6)
Enlarged finger joints	
Yes	205 (88.4)
No	27 (11.6)
Short fingers	
Yes	173 (74.6)
No	59 (25.4)
Elbow extension	
Able	50 (21.6)
Difficult or unable	182 (78.4)
Squat down	
Able	20 (8.6)
Difficult or unable	212 (91.4)

*SD* standard deviation

### Comparison of pre- and posttreatment scores

The pre- and posttreatment scores on the WOMAC and 12-item WHODAS 2.0 are shown in Table 2. Overall, there was statistically significant improvement in the mean scores in all WOMAC domains, and the total score changed from 52.82 (17.87) to 43.22 (20.41) (*p* < 0.001). The 12-item WHODAS 2.0 showed important improvement in all domains except cognition, and the total score changed from 45.32 (21.80) to 39.37 (22.61) (*p* < 0.001). No ceiling or floor effects were detected in the WOMAC questionnaire. However, the WHODAS 2.0 showed a floor effect in the cognition and getting along domains both pre- and posttreatment (Table 2).

**Table 2:** Comparison of the mean (SD) scores and floor and ceiling effects of pre- and posttreatment for each questionnaire (*n* = 232)

Questionnaires	Pretreatment			Posttreatment			Change scores Mean (SD)	t ^a^	*p*-value
	Mean (SD)	% Floor	% Ceiling	Mean (SD)	% Floor	% Ceiling			
WOMAC									
Pain	61.40 (18.86)	0.0	2.6	49.20 (20.93)	1.3	0.9	−12.20 (23.06)	8.06	< 0.001
Stiffness	57.16 (21.92)	0.9	5.6	46.01 (22.77)	2.6	2.2	−11.15 (27.87)	6.09	< 0.001
Function	49.78 (19.24)	0.0	0.4	41.06 (21.76)	0.0	0.0	−8.72 (21.56)	6.16	< 0.001
Total score	52.82 (17.87)	0.0	0.4	43.22 (20.41)	0.0	0.0	−9.60 (20.37)	7.18	< 0.001
WHODAS 2.0									
Cognition	34.90 (26.87)	19.0	4.3	35.83 (29.91)	20.3	7.3	0.93 (32.96)	−0.43	0.669
Mobility	57.74 (28.81)	5.6	12.5	50.27 (29.20)	7.8	9.9	−7.47 (30.89)	3.68	< 0.001
Self-care	46.42 (29.94)	12.5	7.8	37.02 (27.99)	14.7	4.3	−9.40 (32.53)	4.40	< 0.001
Getting along	32.51 (30.32)	30.2	7.3	25.16 (26.10)	37.1	1.7	−7.35 (33.71)	3.32	0.001
Life activities	49.02 (28.61)	9.9	6.9	42.51 (29.10)	13.4	5.6	−6.51 (31.11)	3.19	0.002
Participation in society	51.31 (25.88)	2.2	2.6	45.42 (27.89)	8.2	3.9	−5.89 (29.21)	3.07	0.002
Total score	45.32 (21.80)	0.9	0.0	39.37 (22.61)	2.6	0.0	−5.95 (22.00)	4.12	< 0.001

*SD* standard deviation, *WOMAC* Western Ontario and McMaster Universities Osteoarthritis index, *WHODAS 2.0* World Health Organization Disability Assessment Schedule 2.0 (12-item)

^a^ Paired t-test was used to compare the scores between pre- and posttreatment

### Responsiveness

Table 3 presents the correlation coefficients between the change scores pre- and posttreatment for each outcome measure and the JDI. The change scores for the total WOMAC and all its domains had small to moderate positive associations with the changes in the JDI (r range 0.46 to 0.52, *p* < 0.001). For the correlation between the change scores for the 12-item WHODAS 2.0 and the JDI, weak associations were observed for the total score and most of its domains (r range 0.22 to 0.41, *p* < 0.01); the getting alone domain had no significant association with the JDI (r = 0.04, *p* = 0.509). In addition, the study hypothesis was confirmed (there was a moderate positive correlation between the WOMAC and WHODAS questionnaires, r = 0.57, *p* < 0.001).

**Table 3:** The responsiveness of correlation and ROC curve analyses for each questionnaire^a^

Questionnaires	Correlation analysis		ROC curve analysis			
	Coefficient ^b^	*p*-value	AUC (95% CI)	Optimal cutoff value	Sensitivity	Specificity
WOMAC						
Pain	0.52	< 0.001	0.76 (0.69, 0.83)	22.50	0.68	0.76
Stiffness	0.46	< 0.001	0.74 (0.67, 0.81)	6.25	0.89	0.53
Function	0.45	< 0.001	0.74 (0.67, 0.81)	14.70	0.74	0.68
Total score	0.52	< 0.001	0.77 (0.70, 0.83)	10.41	0.83	0.60
WHODAS 2.0						
Cognition	0.22	0.001	0.64 (0.56, 0.72)	−18.75	0.89	0.31
Mobility	0.41	< 0.001	0.72 (0.65, 0.80)	6.25	0.76	0.58
Self-care	0.26	< 0.001	0.64 (0.56, 0.72)	18.75	0.60	0.67
Getting along	0.04	0.509	0.55 (0.46, 0.64)	18.75	0.43	0.68
Life activities	0.40	< 0.001	0.68 (0.61, 0.76)	−6.25	0.91	0.39
Participation in society	0.38	< 0.001	0.68 (0.60, 0.76)	6.83	0.70	0.58
Total score	0.38	< 0.001	0.71 (0.63, 0.78)	22.31	0.51	0.82

*ROC* receiver operating characteristic, *AUC* area under the curve, *CI* confidence interval, *WOMAC* Western Ontario and McMaster Universities Osteoarthritis index, *WHODAS 2.0* World Health Organization Disability Assessment Schedule 2.0 (12-item)

^a^ The external criterion was the joint dysfunction index (JDI)

^b^ Spearman’s correlation coefficient was used

**Fig. 2:**
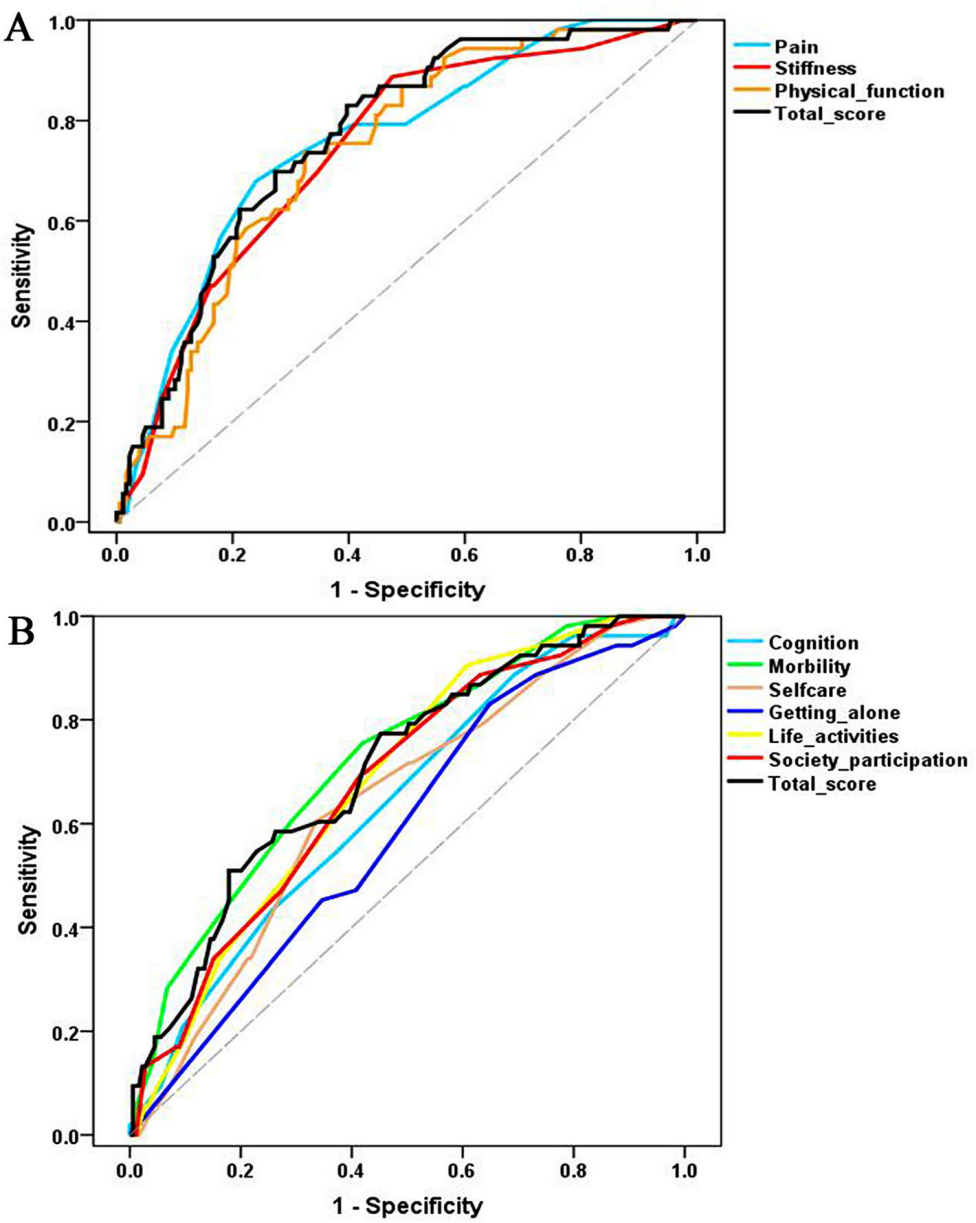
Receiver operating characteristic curves for the change scores for the WOMAC and the 12-item WHODAS 2.0 and their subscales compared with the external criterion of the JDI with an improvement rate ≥ 30%. **a** The WOMAC and its subscales. **b** The 12-item WHODAS 2.0 and its subscales. WOMAC, Western Ontario and McMaster Universities Osteoarthritis index; WHODAS 2.0, World Health Organization Disability Assessment Schedule 2.0; JDI, joint dysfunction index

Based on the JDI, 53 (22.8%) subjects were classified as improved (improvement rate ≥ 30%), whereas 179 (77.2%) were classified as stable or worse (improvement rate < 30%) after the treatment. Table 3 also shows the ROC curve analysis results. The results revealed acceptable AUCs for all WOMAC domains and its total score (values range 0.74 to 0.77). The optimal cutoff value was 10.41 (sensitivity of 83%, specificity of 60%) for the total score. However, only acceptable AUCs for the total score and the mobility domain were obtained for the 12-item WHODAS 2.0 (values of 0.71 and 0.72, respectively), and the optimal cutoff value was 22.31 (sensitivity of 51%, specificity of 82%) for the total score. Fig. 2 shows the ROC curves for the WOMAC, the 12-item WHODAS 2.0 and their subscales.

## Discussion

Self-reported outcome measures for function, pain and disability are widely used as primary endpoints to determine the appropriate intervention strategies for many chronic conditions. Responsiveness is a vital aspect of the quality and suitability of an outcome measure used in clinical research, but it is less frequently studied than reliability and validity [19]. This study was the first evaluation and comparison of the responsiveness of the WOMAC and the 12-item WHODAS 2.0 in KBD patients who received intra-articular injections of HA. To do this, correlation and ROC curve analyses were used as responsiveness statistics using questionnaire data obtained before and after treatment. Overall, the results demonstrated that the WOMAC had a greater degree of responsiveness than did the 12-item WHODAS 2.0 in KBD patients treated with intra-articular injections of HA.

A previous study of OA patients undergoing total knee replacement showed that the WOMAC did not have an important floor or ceiling effect before or after six months of treatment, but a significant ceiling effect was present in the stiffness domain after two years of treatment [29]. In this study, the WOMAC did not have a significant floor or ceiling effect. However, the cognition and getting along domains in the 12-item WHODAS 2.0 had a floor effect both pre- and posttreatment. A significant floor effect in the 12-item WHODAS 2.0 was also found among patients with chronic musculoskeletal pain in a recent study [30]. Higher percentages of floor and ceiling effects were seen in the 12-item WHODAS 2.0 because it has only two items for each domain. Therefore, the 36-item WHODAS 2.0 might be more suitable for evaluating cognition and getting along domains in KBD patients.

Several studies have shown that the WOMAC is responsive to changes in OA patients with different situations [31–33]. A study compared the responsiveness of the WOMAC and generic Short Form-36 (SF-36) in patients with OA of the legs undergoing comprehensive inpatient rehabilitation, and the results showed that both of the measures were able to detect improvement in pain, but the WOMAC was more responsive than the SF-36 in the measurement of function [31]. Another study suggested that the WOMAC physical function domain should be the primary measure of physical function in knee OA patients after comparing the responsiveness of four common patient-reported outcome measures [32]. The responsiveness of the 12-item WHODAS 2.0 in patients with chronic diseases has rarely been studied thus far, although a study showed that the 36-item WHODAS 2.0 had a small level of responsiveness with an effect size (ES) of − 0.34 and standardized response mean (SRM) of − 0.35 in rheumatoid arthritis patients [34].

In this study, significant improvement in the mean scores on the total WOMAC and all of its domains was noted in KBD patients after treatment. These findings were consistent with those of previous clinical trials that used the WOMAC as an outcome measure [16, 17]. Our study also showed significant improvement in the mean scores on the total 12-item WHODAS 2.0 and all its domains, except cognition. The lack of improvement in the cognition domain suggests that the treatment is not related to the patient’s ability to understand and communicate. However, the significant changes in the mean score before and after treatment are dependent on the sample size of the study [19].

The global perceived effect has been commonly used as an external criterion to evaluate properties of other outcome measures [35]. However, we used the JDI in this study because it is an accepted external criterion and has been shown to have good sensitivity for assessing therapeutic efficacy in KBD patients [11]. By conducting a correlation analysis, we found that the change scores for the WOMAC and the 12-item WHODAS 2.0, except for the getting alone domain, were all significantly correlated with the change scores for the JDI; however, the WOMAC had larger correlation coefficients than the 12-item WHODAS 2.0. In addition, the WOMAC and the 12-item WHODAS 2.0 were moderately correlated, in accordance with our hypothesis.

When constructing the ROC curves, an improvement rate of 30% in the JDI was used to dichotomize patients into two groups: improved and unimproved (stable or worse) patients. Considering this criterion, the AUCs were greater than 0.70 for all WOMAC domains and its total score, thus indicating that the WOMAC can reliably discriminate between improved and unimproved KBD patients after treatment. A recent study also showed that the WOMAC had a satisfactory discriminatory ability in subjects undergoing rehabilitation following hip fracture [36]. However, the AUCs of most domains in the 12-item WHODAS 2.0 did not reach the recommended 0.7 standard, which suggested inadequate responsiveness of this questionnaire for the KBD patients in the present study.

This study has some limitations. First, we did not formulate detailed a priori hypotheses regarding the expected changes before and after the intervention regarding the construct approach of responsiveness evaluation recommended by the COSMIN guidelines. Moreover, another important limitation is the duration of the follow-up period. The patients were given intra-articular injection once per week for three weeks, and the last interview was carried out after the treatment. This period may not have been sufficiently long to detect a meaningful change. Therefore, long-term follow-up for KBD patients following treatment is necessary in future studies.

## Conclusions

In summary, this study has shown that the WOMAC is more responsive than the 12-item WHODAS 2.0 to changes over time in KBD patients receiving intra-articular injections of HA. Thus, the WOMAC is a preferable questionnaire for use as an outcome measure in assessing disability associated with KBD. Our findings provide useful information that can aid clinicians or researchers in selecting appropriate outcome measures in future clinical settings for KBD patients.

## Data Availability

The datasets used and/or analyzed during this study are available from the corresponding author on reasonable request.

## Abbreviations

AUC: Area under the curve
CDC: Centers for Disease Control and Prevention
ES: Effect size
HA: Hyaluronic acid
ICF: International Classification of Functioning Disability and Health
JDI: Joint dysfunction index
KBD: Kashin-Beck disease
OA: Osteoarthritis
ROC: Receiver operating characteristic
SD: Standard deviation
SRM: Standardized response mean
VAS: Visual analog scale
WHODAS: World Health Organization Disability Assessment Schedule
WOMAC: Western Ontario and McMaster Universities Osteoarthritis index
WHO: World Health Organization
